# Reinfection as a central constraint on tuberculosis vaccine development

**DOI:** 10.3389/fimmu.2026.1807344

**Published:** 2026-04-21

**Authors:** Pere-Joan Cardona

**Affiliations:** 1 Experimental Tuberculosis Unit (UTE), Institut de Recerca Germans Trias i Pujol (IGTP), Badalona, Spain; 2 Microbiology and Genetics Department, Universitat Autònoma de Barcelona, Bellaterra, Spain; 3 Servei de Microbiologia, Laboratori Clínic de la Metropolitana Nord (LCMN), Hospital Universitari Germans Trias i Pujol (HUGTiP), Badalona, Spain; 4 Centre de Medicina Comparativa i Bioimatge de Catalunya (CMCiB), Badalona, Spain; 5 Centro de Investigación Biomédica en Red de Enfermedades Respiratorias (CIBERES), Madrid, Spain

**Keywords:** C3HeB/FeJ, immunity to *Mycobacterium tuberculosis*, multiple consecutive infections, prevention of disease, reinfection, TB vaccines, tuberculosis, vaccine evaluation models

## Abstract

Tuberculosis (TB) vaccine development is hindered by the absence of validated correlates of protection, the low proportion of infected individuals who progress to disease, and the pervasive impact of reinfection in endemic settings. Although several candidates have advanced clinically, only M72/AS01E has demonstrated prevention of disease in adults. These limitations underscore the need to reassess both vaccine objectives and preclinical evaluation strategies. Epidemiological, immunological, and experimental evidence indicates that natural *Mycobacterium tuberculosis* infection can provide substantial protection against disease progression in immunocompetent adults through lung-localized adaptive immunity. However, this protection is anatomically restricted and initially eroded by repeated endogenous reinfection. In high-transmission environments, repeated exposures simultaneously increase bacillary burden while boosting host immune responses, thereby reducing the incremental benefit of vaccination. An *in silico* multiple consecutive infection (MCI) model predicted that successive infections would produce a cumulative rise in pulmonary bacillary load reaching a plateau, together with an exponential decline in BCG efficacy. This prediction was subsequently validated experimentally in the C3HeB/FeJ murine model, which develops neutrophil-rich, liquefaction-prone lesions resembling human active TB. Repeated daily infections reproduced the anticipated increase in bacillary burden but paradoxically reduced the proportion of neutrophilic exudative lesions, reflecting modulation of inflammatory pathology. Under MCI conditions, BCG conferred only marginal additional protection compared with single infection. Collectively, these findings identify reinfection as a central biological constraint on prophylactic TB vaccines and support incorporating MCI models into preclinical pipelines to enhance predictive value and prioritize prevention of disease.

## 1 Introduction

Tuberculosis (TB) vaccine development remains a formidable challenge, largely due to the absence of validated correlates of protection (1) and the low proportion of infected individuals who progress to active disease (2, 3). Recent clinical developments have yielded only modest results, ranging from the lack of prevention of disease (POD) by MVA85A (4) to the failure to prevent infection (POI), measured as IGRA conversion, by H4:IC31 (5), and the lack of prevention of recurrence (POR) by H56:IC31 (6). To date, M72/AS01E is the only candidate to have demonstrated partial efficacy, achieving a 54% reduction in disease among IGRA-positive adults (7), confirming a 49.7% efficacy with sustained immunogenicity after the 36-month final analysis (8).

While definitive Phase 3 efficacy data for the M72 pivotal registration trial (9) are still pending, MTBVAC has completed Phase 2a evaluation in South African infants, demonstrating safety and immunogenicity and informing dose selection for further development (10, 11). In this evolving vaccine landscape, it remains timely to revisit the impact of reinfection, which is essential for assessing the biological and epidemiological constraints faced by prophylactic TB vaccines targeting POI, POD, and POR.

## 2 Does *Mycobacterium tuberculosis* infection provide protection against TB disease?

In 1938, observational studies among nursing students at Oslo Hospital revealed that individuals with prior *Mycobacterium tuberculosis* (Mtb) infection exhibited a 97% protection against active TB, markedly higher than that observed in BCG-vaccinated individuals (12, 13). Despite later concerns regarding potential confounding factors (14), these findings highlighted the strong protective effect of natural infection in a high-risk population.

In immunocompetent adults, progression to pulmonary TB is largely an inflammatory process occurring preferentially in the upper lobes, which constitute roughly 20% of the lung parenchyma and provide a permissive environment for neutrophil recruitment and extracellular bacillary growth (15). In the remaining lung tissue, infection typically induces a robust adaptive immune response characterized by rapid expansion of antigen-specific lymphocytes capable of activating infected alveolar macrophages. This lung-localized, mucosal immunity is generally more effective than immunity elicited through systemic immunization (16).

In contrast, pediatric TB presents a distinct immunopathological landscape. Ongoing lung development, including a dense and remodeling capillary network surrounding alveoli, facilitates hematogenous dissemination, accounting for the high risk of extrapulmonary disease in children and the protective benefit of early BCG vaccination (17).

Endogenous reinfection following primary infection can progressively expand the proportion of lung tissue at risk, explaining why the highest incidence of disease occurs within the first year after infection and declines thereafter (2, 3, 18).

## 3 Are we using the correct experimental models to test vaccine candidates?

The protection value of reinfection was demonstrated more than twenty years ago in an experimental model of murine TB, highlighting its role in the development of TB, and the need for a novel vaccine to perform better than both vaccination with BCG and immunity induced by natural infection (19).

**Figure 1:**
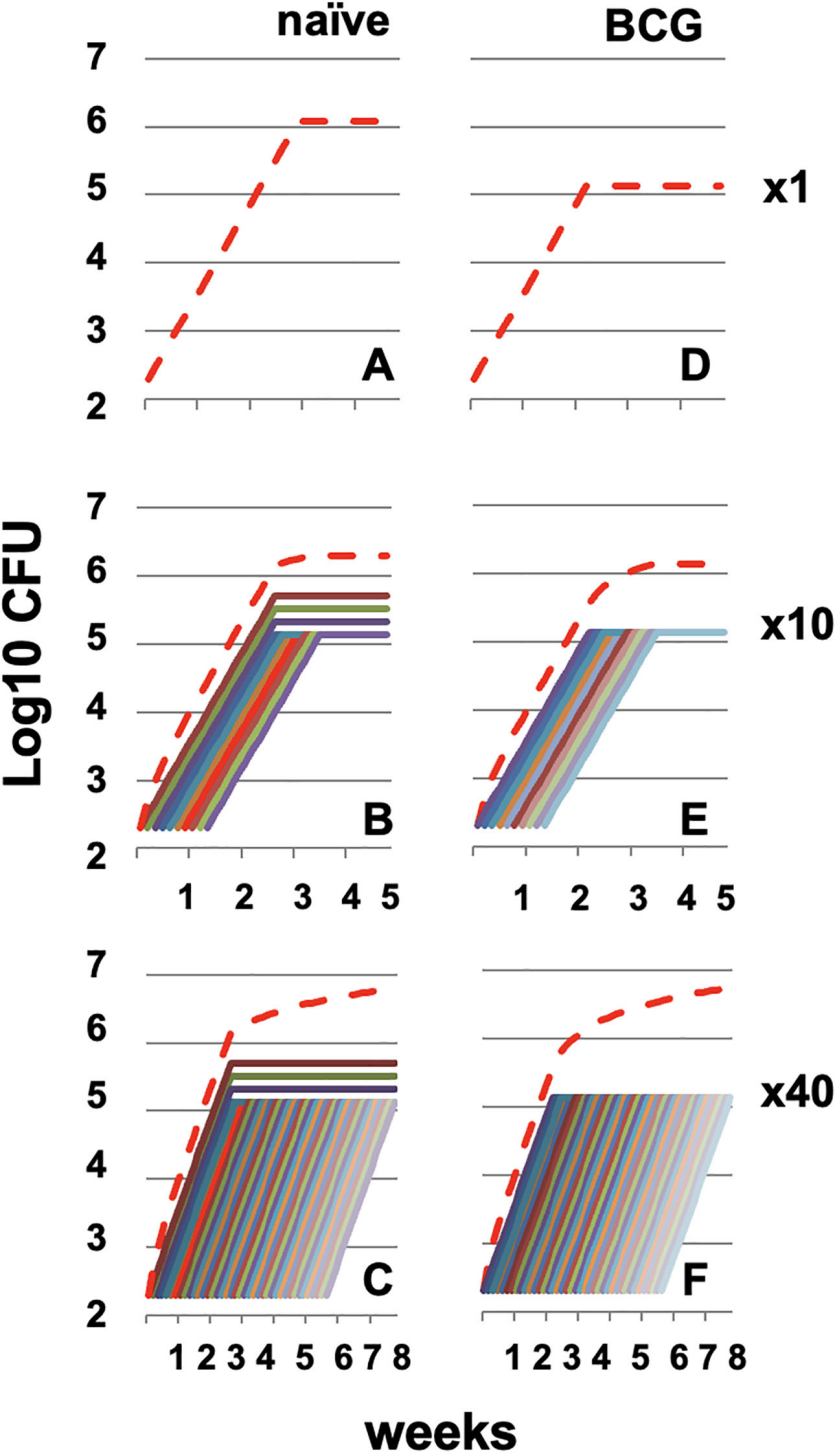
Evolution of the bacillary load in the “in silico” model of single or multiple consecutive infections (MCI). Progression of the bacillary concentration in both naïve **(A–C)** and BCG-vaccinated mice **(D–F)** after single infection **(A, B)** or MCI with 10 (x10) or 40 (x40) consecutive infections. The red line represents the sum of the bacillary load at all individual infection sites. The growth model follows an exponential equation (*N* = *No* * 2^(t/2.3)^ where *No* is the initial dose (100 CFUs) and t is the time in days. Obtained from (20).

“In silico” modeling of multiple consecutive infections (MCI) in mice demonstrated that repeated exposure to Mtb results in a cumulative increase in pulmonary bacillary burden, although this increase plateaus with higher numbers of consecutive infections. Under these conditions, the protective efficacy of BCG declines exponentially, providing minimal additional reduction in bacterial load after repeated exposure (20) (**Figure 1**). This model reveals that although reinfection increases bacillary burden and generates new infectious foci, it simultaneously boosts host immune responses. From an immunological perspective, BCG vaccination may therefore resemble a controlled reinfection that enhances immunity without increasing pulmonary bacillary load.

**Figure 2:**
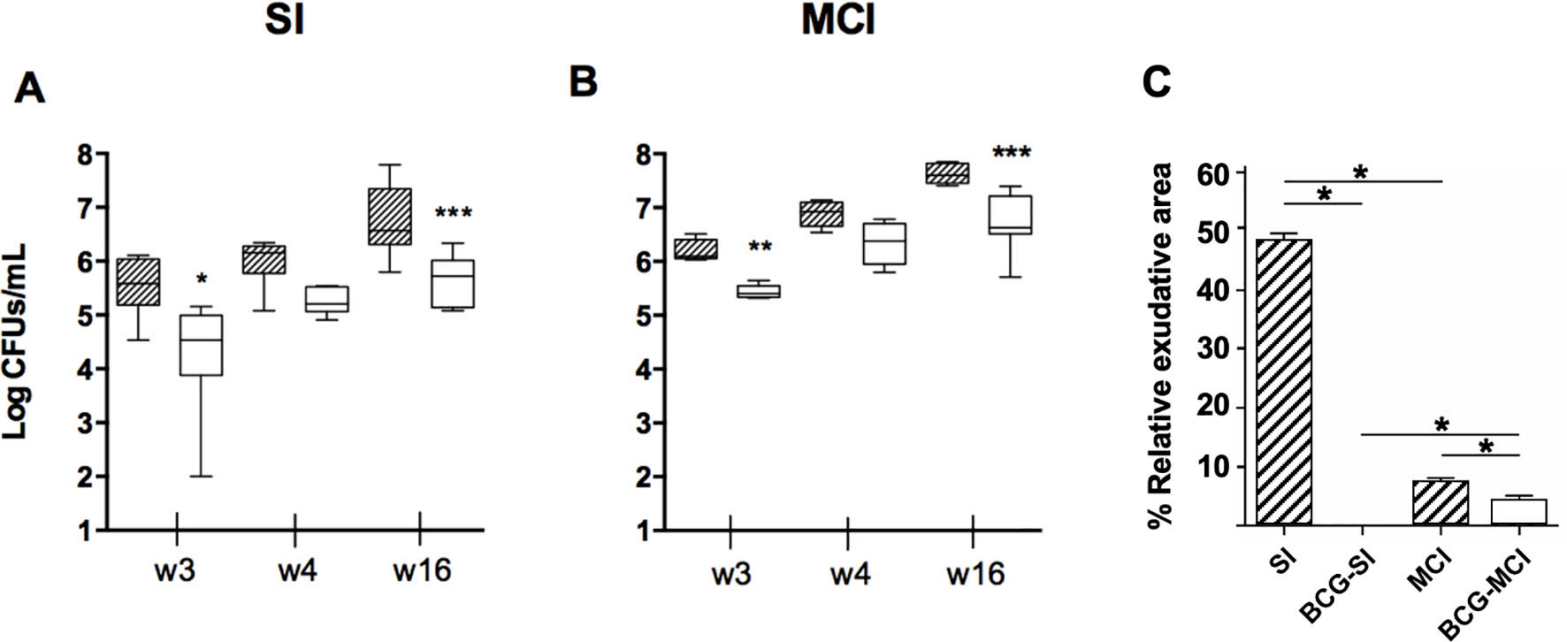
Bacillary load progression and quality of the lesions. Bacillary load (in CFUs/mL) is shown at different end time points (w3, w4, and w16). Each panel compares sham (hatched) and BCG vaccinated (clear) groups for single infection (SI) **(A)** and 8 daily multiple consecutive infections (MCI) **(B)**. Box and whiskers plots show the minimum, first quartile, median, third quartile and maximum values. *p < 0.05, **p < 0.01, ***p < 0.001; Mann–Whitney test. Picture **(C)** shows the relative percentage of exudative lesions at week 16. *p < 0.05, Mann–Whitney test. Obtained from (24).

Validation was performed using the C3HeB/FeJ mice, that gives information not only on the control of the bacillary load, but also in the capacity to reduce neutrophil infiltrated lesions, with a rapid progression, which resemble liquefacted human like lesions, a proxy of development of active TB (21–23). As predicted by in silico modeling, daily consecutive infections over eight days resulted in the expected 1-log increase in bacillary load while global lung neutrophilic infiltration was reduced by 40%. Of note BCG vaccination contributed a discrete reduction (5%) in exudative lesions in the context of MCI, whereas this effect was dramatic after a single infection (24) (**Figure 2**).

Consequently, in high-incidence settings with intense reinfection pressure, the incremental protective value of BCG can decrease, as reinfection itself both drives immune activation and modulates pathological inflammation.

## 4 Can infection be prevented?

Mtb infection begins when inhaled bacilli reach the alveoli, infect alveolar macrophages, and replicate until host cell necrosis occurs (25). This death macrophage is replaced in first instance by an interstitial one (26), which also is unable to stop the bacillary growth. Meanwhile, bacilli spread through other alveoli, infecting other alveolar macrophages. Subsequent recruitment of interstitial macrophages, monocytes, and neutrophils leads to local inflammation, disruption of alveolar homeostasis, and lymphatic dissemination to regional lymph nodes, where adaptive immunity is initiated and detected by tuberculin skin testing or IGRA conversion (27).

Importantly, this process can be interrupted at multiple stages by innate mechanisms. Most inhaled bacilli fail to reach the alveoli and are cleared by mucociliary transport. Within the alveolar space, lung surfactant hydrolases dramatically modify *M. tuberculosis* envelope, resulting in a significant decrease (60–80%) in *M. tuberculosis* association with, and intracellular growth of the bacteria within, human macrophages (28). Trained innate immunity, as observed in BCG-vaccinated IGRA-negative household contacts, can further enhance early bacillary control (29).

Thus, while “early” infection can be prevented, this protection is mediated primarily by innate immunity. Adaptive Th1 responses arise only after infection is established, rendering durable prevention of infection through classical vaccination strategies biologically challenging. The contribution of antibodies remains uncertain (30, 31), particularly given the restricted access of plasma immunoglobulins to the alveolar space.

In the context of repeated exposure, classical systemic Th1 responses are unlikely to constitute sufficient correlates of protection. Within the MCI framework, more biologically meaningful readouts may include lung-resident memory T cell responses, early innate macrophage antimicrobial programs, stability of bacillary plateau under repeated challenge, and reduced neutrophil-dominated exudative pathology. Protection against disease progression in endemic settings may therefore depend less on the magnitude of systemic IFN-γ responses and more on the capacity to modulate local inflammatory architecture and contain newly seeded pulmonary foci.

## 5 Can recurrence be prevented?

Individuals successfully treated for active TB have an approximately sevenfold increased risk of developing recurrent disease following reinfection (32). Thus, POR through vaccination might be one of the major challenges. At this point it is important to understand what it means to be cured in TB. According to the WHO this is linked to a sustained negativity of the sputum culture and/or a good compliance of the treatment. This wide strategy allows up to 85% of efficacy (33), a fact that also depends on the severity of the TB at the time of diagnosis, thus there is some margin for reactivation.

The difficulty of achieving POR has recently been illustrated in a Phase 2b trial of H56:IC31 in successfully treated, HIV-negative adults, which failed to demonstrate a reduction in recurrent TB despite achieving acceptable immunogenicity (6). This negative result provides empirical support for the biological constraints discussed above. In high-incidence settings, recurrent disease is dominated by exogenous reinfection rather than simple reactivation and is further compounded by genetic susceptibility and residual lung damage following prior disease (34). Because reinfection is often unavoidable under these conditions, vaccine-induced boosting of systemic adaptive responses may be insufficient to counteract repeated bacillary seeding. Consequently, POR vaccines would likely require efficacy levels—and possibly a quality of lung-localized immune control—exceeding those needed for primary disease prevention and surpassing what current subunit strategies have achieved.

## 6 Conclusion: What added value should a TB vaccine provide?

Natural Mtb infection can induce substantial protection against progression to active disease in immunocompetent adults through lung-localized adaptive immunity; however, this protection is incomplete, anatomically constrained, and initially eroded by endogenous reinfection. In children, the inherent risk of dissemination explains the continued benefit of early BCG vaccination. In endemic settings, repeated reinfection both boosts immunity and increases bacillary burden, thereby limiting the incremental benefit of vaccination. This fact highlights the need of including MCI models to test vaccine candidates in a more robust manner. Early control of infection relies largely on innate immune mechanisms, while adaptive immunity emerges only after infection is established, challenging the feasibility of sustained POI as a vaccine endpoint. Finally, the marked susceptibility of cured individuals to reinfection highlights POR as a critical—and particularly challenging—objective for next-generation TB vaccines.

## Data Availability

The original contributions presented in the study are included in the article/supplementary material. Further inquiries can be directed to the corresponding author.
